# Glycans in Virus-Host Interactions: A Structural Perspective

**DOI:** 10.3389/fmolb.2021.666756

**Published:** 2021-06-07

**Authors:** Nathaniel L. Miller, Thomas Clark, Rahul Raman, Ram Sasisekharan

**Affiliations:** ^1^ Harvard-MIT Division of Health Sciences and Technology, Massachusetts Institute of Technology, Cambridge, MA, United States; ^2^ Department of Biological Engineering, Massachusetts Institute of Technology, Cambridge, MA, United States; ^3^ Koch Institute for Integrative Cancer Research, Massachusetts Institute of Technology, Cambridge, MA, United States

**Keywords:** glycoepitope, glycans, 2G12, topology, virus

## Abstract

Many interactions between microbes and their hosts are driven or influenced by glycans, whose heterogeneous and difficult to characterize structures have led to an underappreciation of their role in these interactions compared to protein-based interactions. Glycans decorate microbe glycoproteins to enhance attachment and fusion to host cells, provide stability, and evade the host immune system. Yet, the host immune system may also target these glycans as glycoepitopes. In this review, we provide a structural perspective on the role of glycans in host-microbe interactions, focusing primarily on viral glycoproteins and their interactions with host adaptive immunity. In particular, we discuss a class of topological glycoepitopes and their interactions with topological mAbs, using the anti-HIV mAb 2G12 as the archetypical example. We further offer our view that structure-based glycan targeting strategies are ready for application to viruses beyond HIV, and present our perspective on future development in this area.

## Introduction

Glycosylation is a common post-translational modification of all proteins where complex glycans are attached to specific amino acids such as Asn (in the case of N-linked glycans) and Ser or Thr (in the case of O-linked glycans). Consequently, complex glycans are displayed on surfaces of viruses and host tissues and cells and play multidimensional roles in pathogen-host interactions (Van Breedam et al., 2014; Stroh and Stehle, 2014; Smith and Cummings, 2014; Raman et al., 2014). The structural diversity of these glycans is governed primarily by the complex non-template driven biosynthetic machinery of the host cell involving several enzymes that show tissue-specific expression patterns (Lowe and Marth, 2003; Taylor and Drickamer, 2003; Varki et al., 2008). Additionally, glycan diversity is also influenced by the spatial distribution of the glycosylation sites on the protein surface, wherein glycan clustering can result in steric hindrance of glycan processing enzymes (Bonomelli et al., 2011).

**FIGURE 1:**
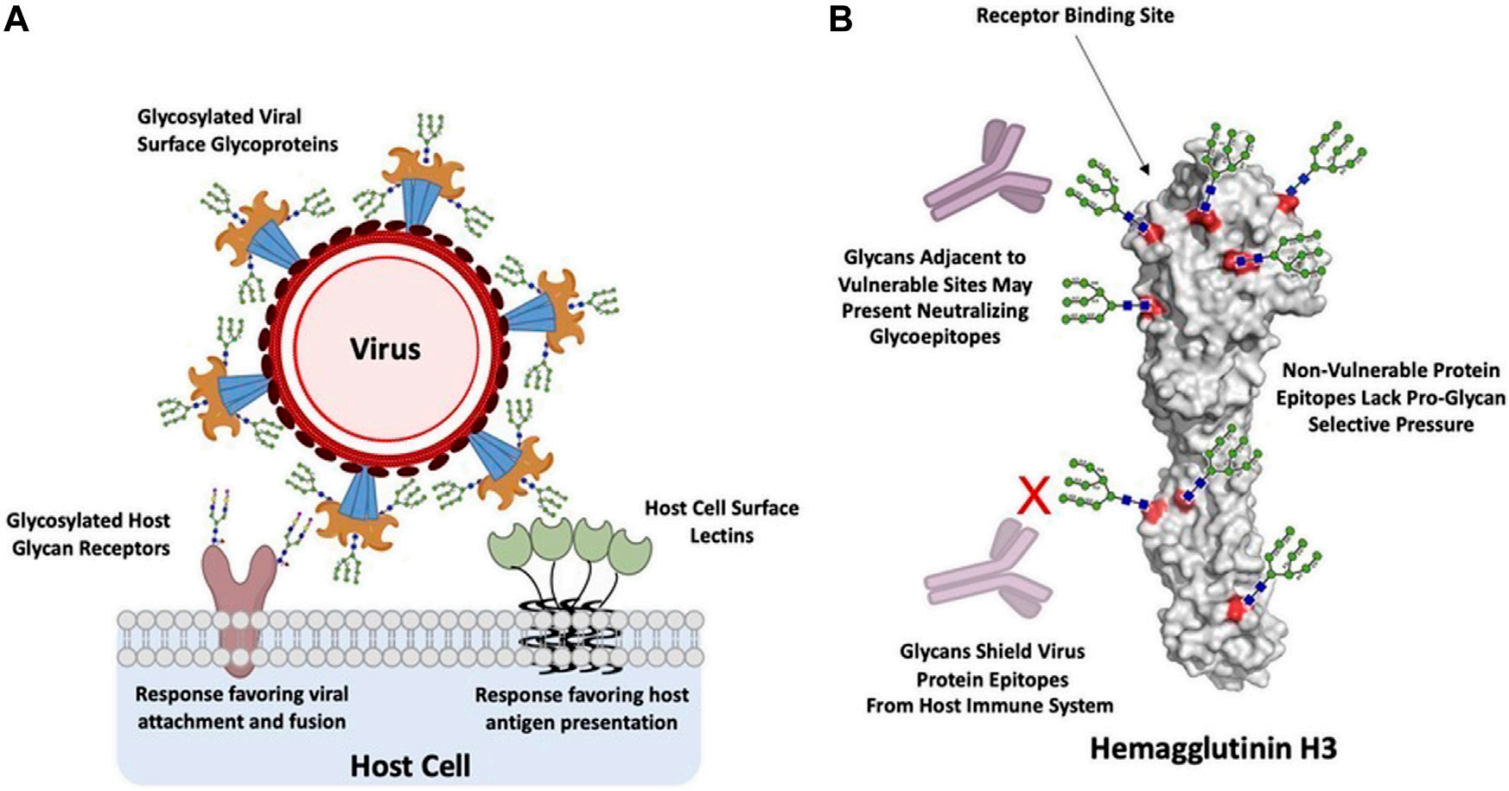
Glycan interactions between host and virus. **(A)**, glycan interactions between host cells and viruses are predominantly mediated by host or virus glycoproteins and their complementary host or virus glycan binding proteins. These binding events trigger processes that favor either viral attachment and fusion or host recognition and response. **(B)**, Hemagglutinin H3 from Influenza A Wyoming/3e/2003, depicted with Man9 glycans and theoretical mAbs for illustrative purposes. Glycans shield vulnerable protein epitopes from neutralizing mAbs, such as the receptor binding site, while non-vulnerable protein epitopes lack selective pressure for glycan shielding. However, these glycans can also present targets as glycoepitopes. Generated in PyMOL from PDB: 6BKN (Wu et al., 2018).

Glycans on host cell or tissue surfaces serve as attachment factors, co-receptors, or primary receptors that are specifically recognized by the viral surface glycoproteins. For example, complex glycans terminated by α2-3- or α2-6-linked sialic acid (N-acetyl neuraminic acid) act as receptors for several different viruses (Stencel-Baerenwald et al., 2014). Linear sulfated glycosaminoglycans such as heparan sulfate act as co-receptors for a variety of viruses (Wadstrom and Ljungh, 1999) including dengue virus (Chen et al., 1997; Artpradit et al. 2013), hepatitis C virus (Baumert et al., 2014), and foot-and-mouth disease virus (Fry et al., 1999). The predominant display of specific glycan motifs on surfaces of different cells and tissues contributes to the host restriction and cell/tissue tropism of the virus (de Vries et al., 2010). As an example, the human upper respiratory epithelial surface predominantly displays sialylated glycan receptors terminated by α2-6-linked sialic acid and these receptors are specifically recognized by hemagglutinin glycoprotein (HA) on the surface of influenza A viruses that are known to infect and transmit *via* respiratory droplets in humans (Neumann and Kawaoka, 2006; Malik, 2009; Ge and Wang, 2011; Raman et al., 2014). On the other hand, when influenza A viruses that are commensal or epizootic in birds infect humans, they typically affect deep lung and other tissues that predominantly display sialylated glycan receptors terminated by α2-3-linked sialic acid and are unable to transmit efficiently *via* respiratory droplets. In other cases, host cells also display proteins that specifically bind to glycans on pathogen surfaces thereby acting as key receptors for glycan-mediated viral infection (Figure 1A).

The glycans displayed on viral surfaces are post-translational modifications of viral surface proteins such as envelop proteins in flaviviruses (i.e., dengue and zika viruses) and glycoprotein spikes (i.e., influenza A, Ebola, and SARS-CoV-2 viruses). These glycans are added to the viral glycoproteins as a part of the host-cell glycan biosynthesis during viral replication in the host. In many cases, glycosylation sites on viral surface proteins are highly conserved since the glycans at these sites critically maintain the stability of these proteins and the viral particles as a whole (Wagner et al., 2002; Zhang et al., 2015). In addition to maintaining the stability of the viral particles, these glycans also play key roles in mediating infection of host cells by certain viruses such as dengue and Ebola viruses through specific interactions with glycan-binding proteins (such as C-type lectins) displayed on the host cell surface (Klimstra et al., 2003; Mondotte et al., 2007; Pipirou et al., 2011; Van Breedam et al., 2014).

The complex glycans on the viral surface also play a key role in host immune response to counter the viral infection. While glycan-binding proteins anchored on surfaces of host cells such as dendritic cells (DCs) play a role in viral entry, they also play a dual role to enhance antigen presentation and processing for adaptive immune response (Van Breedam et al., 2014). Sites of N-linked glycosylation are often positively selected during evolution of the virus in human host to increase glycans on the viral surface thereby presenting glycans that mimic self-antigens and mask the underlying protein epitope which in turn permits the virus to evade host immune response. In other cases, particularly with HIV, the clustering of glycosylation sites on the gp120 surface glycoprotein presents novel glycoepitopes that do not mimic self-antigens and therefore lead to potent neutralization by antibodies that target these novel epitopes across a broad spectrum of viral strains (Doores, 2015).

In this perspective, we review the role of viral glycan-host interactions, emphasizing the mechanisms through which glycan structure drives virus immune shielding or immunogenicity. We discuss the role of viral surface glycans in the context of presenting glycoepitopes for the host immune system and examine the various categories of glycoepitopes according to their structure and interaction with glycan-binding agents and antibodies. In particular, we examine a class of high-mannose glycoepitopes we refer to as topological glycoepitopes, using the anti-HIV mAb 2G12 as the archetypical example. Finally, we provide our perspective on fruitful directions for future investigation of topological glycoepitopes, and the rational engineering of antibodies targeting them.

## Viral Surface Glycosylation and Host Immune Response

During interactions with host immunity, glycans fall along a spectrum from immunogenic to immune shielding depending on their species, location on the viral glycoprotein, and topological arrangement relative to one another. A major function of N-linked glycosylation on virus glycoproteins is shielding antigenic sites from viruses, as is particularly well established in the cases of Influenza A Tate et al. (2014) and HIV Mascola and Montefiori (2003). This adaptation has a clear structure function-relationship, in which glycans are commonly added adjacent to major functional sites that tend to be the targets of neutralization by host antibodies, thus focusing immune responses away from these sensitive epitopes Bajic et al. (2019), such as for the Flu head proximal to the functional RBS (Figure 1B). In another example, the functional attachment and fusion domains of the Ebola glycoprotein are protected by a glycan cap, which is hypothesized to prevent neutralizing host antibody responses directed against this major functional target (Lee et al., 2008). Similarly, the Epstein-Barr virus is protected by a glycan shield, which has been shown to additionally impair T cell responses directed against infected cells (Gram et al., 2016). The structural basis for these cases of shielding is rather straightforward—glycans sterically prevent host immune recognition of protein epitopes at these sites. Indeed, in a recent investigation of the SARS-CoV-2 glycan shield, Watanabe et al. leveraged extensive site-specific glycan analysis of the glycoproteins of HKU1, SARS, MERS, LASV, H3N2, HIV-1 Env, SIV Env to highlight a correlation between glycoprotein glycan shield density, oligomannose content, and viral immune evasion (Watanabe et al., 2020). Further evidence for this effect can be observed in the viral evolution of Influenza A, which has been shown to add N-glycan sites every 5–7 years Altman et al. (2019) as a result of selective pressure on sensitive protein epitopes in a pattern known as a “glycan clock.” In contrast, glycans decorating less immunogenic regions tend to be conserved, and may contribute beneficially through other mechanisms such as stabilizing the fusion event (Ohuchi et al., 1997).

While immune-shielding glycans are beneficial for immune evasion, they do not solely accumulate over time. Glycosylation bears fitness costs due to increased metabolic resources required for replication and the potential disruption of glycoprotein function. Therefore, immune-shielding glycans may be maintained or eventually lost, often with significant impact on host-virus interaction. Spontaneous glycan loss proximal to the receptor binding site drove virulence of 2009 H1N1 (Wei et al., 2010). This finding was supported by a subsequent study in mice that found evidence that a similar event drove the virulence of the 1918 pandemic H1N1 (Sun et al., 2013). However, these fitness tradeoffs vary significantly across viruses. As compared to the global evolution of Flu, HIV’s glycan shield provides a persistent shielding benefit, both within chronically-infected hosts Coss et al. (2016) and evidently throughout global evolution.

Further, glycan structure and function are dependent on neighboring glycan context for a given glycosylation site. As N-glycan sites accrue in close proximity to one another over the course of viral evolution, their clustering tends to result in oligomannosylation of the N-glycans at nearby sites. The structural mechanisms by which clustered N-glycan sites result in high mannose species have been discussed elsewhere (Behrens and Crispin, 2017). In brief, the effect is likely driven by high glycan density sterically limiting α-mannosidase mediated glycan trimming during glycan processing in the Golgi. This effect appears to be conserved across viruses, and is best defined for HIV in which it is conserved across a range of HIV clades and production systems (Bonomelli et al., 2011). Further, in a recent examination of glycan species heterogeneity for various recombinant Flu vaccine production systems, the FDA found significant glycan variance for the less clustered and non-oligomannose glycans of H1N1 across egg, insect, or eukaryotic cell production systems, yet found that high oligomannose content was preserved across these production systems for the more clustered glycans of H3N2 (An et al., 2019). Still, other mechanisms may also contribute to the presence of high-mannose glycans on certain viruses in certain host contexts. A recent ferret study found that Influenza infection of lung cells induces high-mannose N-glycans on host proteins in a manner dependent on activation of the unfolded protein response (UPR) *via* IRE1 (Heindel et al., 2020). It is plausible that this pathway may also contribute to oligomannosylation of glycans on viral glycoproteins, and this would likely be independent of N-glycan clusters but rather a property of viral infection itself activating the UPR. Importantly, high-mannose glycans are relatively rare on mature host cells, and are typically only presented within the body at high frequency on stem cell membranes (An et al., 2012). As such, high-mannose glycans are better recognized as foreign by the host, and they themselves may become the target of immune responses, serving as “glycoepitopes.” The canonical example of this recognition occurs on HIV gp120 (Doores et al., 2010), and will be discussed in detail in the following section.

## Defining Glycoepitopes on Viral Surfaces

**FIGURE 2:**
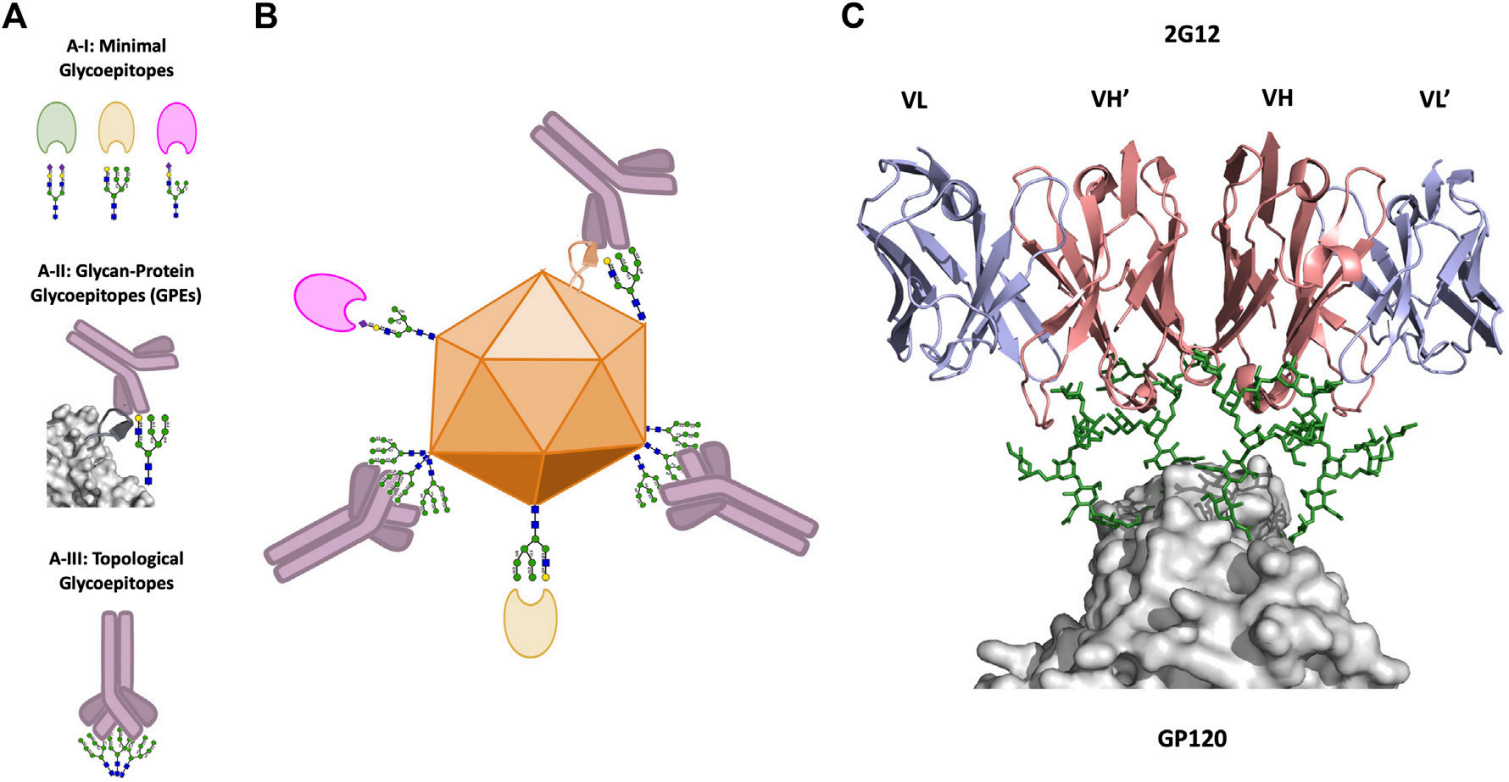
Viral glycoepitopes. **(A)**, Viruses present three types of glycoepitopes for targeting by the host immune system. 2A–I: Minimal glycoepitopes consist of glycans which are effectively recognized specifically by host lectins. 2A–II: Glycoprotein glycoepitopes (GPEs) include both glycan and peptide constituents, and are most effectively neutralized by Y-shaped mAbs. 2A–III: Topological glycoepitopes exist at clusters of high-mannose glycans, and are flexibly recognized by X-shaped mAbs such as 2G12. **(B)**, illustration of the three types of glycoepitopes and their preferred mode of host-recognition. **(C)**, crystal structure of the interaction between the canonical topological glycoepitope on HIV and the X-shaped mAb 2G12 (PDB: 6OZC, Seabright et al., 2020). Heavy chains are shown in red, light chains are shown in blue, glycans are shown in green, and the virus protein surface is shown in gray (PyMol, 2019).

While we have thus far discussed glycans predominantly as beneficial to viral fitness in interactions with the host immune system, glycans may also serve as immune targets (glycoepitopes). We next review three broad types of glycoepitopes, categorized according to the structural-basis for their recognition event (Figure 2A). These types are: 1) *minimal glycoepitopes*: glycoepitopes recognized chiefly based on their glycan species, typically most effectively by lectins or pattern recognition receptors (Figure 2A-I); 2) *glycan-protein glycoepitopes (GPEs)*: glycoepitopes formed by combinations of glycan and peptide epitopes and recognized by antibodies (Figures 2A-II); and 3) *topological glycoepitopes*: glycoepitopes occurring at N-glycan clusters with a predisposition for oligomannose, and recognized by unconventional antibodies based on their glycan topology without regard for protein sequence (Figures 2A-III). We focus primarily on the third class, using the anti-HIV mAb 2G12 as the canonical topological mAb to explore the unique features of topological glycoepitopes.

### Minimal Glycoepitopes

First, glycoepitopes defined primarily according to the glycan species represents the broadest group, with extent of structural characterization depending on the specific biological context and availability of lectins binding these species, such as mannose-binding lectin and its mannose target (Ji et al., 2005) (Figures 2A-I). These glycoepitopes are recognized by host pattern recognition receptors (PRRs) to activate a variety of host immune responses, typically *via* opsonization (Brown et al., 2018). These recognition events occur across microbes, including viruses, bacteria, and fungi, and their structural basis has been reviewed extensively elsewhere (Wesener et al., 2017). We note that many agents binding this class of glycoepitope have significant recognition breadth, and may also engage the following two groups of glycoepitopes, albeit usually with reduced specificity and affinity, which has tended to complicate therapeutic development of these agents. Recent engineering efforts have sought to optimize these properties in pursuit of enhanced clinical applicability Hamorsky et al. (2019); Covés-Datson et al. (2020), and thus represent a creative and orthogonal approach to the GPE and topological glycoepitope targeting strategies we discuss next.

### Glycan-Protein Glycoepitopes

Second, glycoepitopes may be defined by a combination of glycan and protein, with targeting primarily driven by antibodies (Figures 2A-II). Human IgG and IgM serum have high concentrations of anti-glycan antibodies targeting a diverse range of carbohydrate antigens, including antibodies against both xeno-antigens [e.g. Gal(\alpha1-3)Gal] Bovin et al. (2012) and allo-antigens (e.g., ABO blood group associated antigens) (Yamamoto et al., 1990). Many of these antibodies recognize di- or tri-saccharide motifs, but fail to recognize the same motif in the context of a longer complex glycan, a masking effect that is hypothesized to limit autoimmune response Bovin et al. (2012), although this effect cannot necessarily be attributed to size alone (Huflejt et al., 2009).

Anti-glycan antibodies can be divided into *natural* and *antigen-dependent* antibodies. Natural antibodies exist without any antigenic stimulation or T-Cell dependence, while *antigen-dependent* antibodies are generated and matured specifically in response to antigenic stimuli. The mechanism of *antigen-dependent* immune response to various glycoepitopes (glycoprotein, glycolipid, oligosaccharide) is not fully understood, due to their heterogeneous nature and their relationship to the well-established T-cell response for peptide antigens (Kappler and Hennet, 2020). However, research into the subset of antibodies targeting glycan-protein epitopes (GPEs) on glycoproteins has provided structural evidence of antigen-mediated antibody evolution. Identifying the critical structural mechanisms of binding for these GPE targeting mAbs can be used to optimize both therapeutic mAb design and vaccine design.

Immune response to the HIV env glycoprotein, due to its extensive glycan shield, has generated broadly neutralizing HIV antibodies targeting several distinct glycoepitopes (Burton et al., 2009; Pejchal et al., 2011; Walker et al., 2011; Sanders et al., 2013; Falkowska et al., 2014). The glycan-dependence of these antibodies varies from fully glycan-dependent with no associated protein interface (2G12, discussed in the next sub-section on topological glycoepitopes), to protein and glycan-dependent (PG9, PG16, and PGT121-128) (Burton et al., 2009; Pejchal et al., 2010). Examining the similarities and differences of GPE mAbs (“PGT121-124”) (Walker et al., 2011; Garces et al., 2014) illustrates key aspects of canonical paratope-GPE interaction: broadly similar paratope structure, divergent mechanisms of interaction after affinity maturation, and glycan-species dependent binding.

Antibodies PGT121-124 all share a canonical paratope structure that has properties characteristic of GPE-binding mAbs. An abnormally-long H3 loop in association with the light chain forms an elongated face that interacts with the GPE glycan at residue N332 (Julien et al., 2013), and a “GDIR” motif (residues 324-327) that interacts with the protein components of the GPE (Garces et al., 2014). Further, the interactions with GDIR and N332 are well-defined in all the mAbs in this lineage and fixed early in the maturation process from germline (Garces et al., 2015). That is, a long H3 loop to penetrate the glycan shield while also interacting with the GPE glycan, a peptide-binding motif, and a stabilizing glycan are critical attributes of this epitope-paratope interaction that may characterize other GPE epitope-paratope interactions as well. PGT121-124 have similar high-level structural characteristics, but differ significantly in terms of their final paratope amino acid sequences due to diverging paths of natural affinity maturation. In addition to the “elongated face,” the PGT121-124 also contain an “open face” that is comprised of residues in CDRH1, CDRH2, CDRH3 (Garces et al., 2014). Somatic hypermutation during affinity maturation in this open face leads to the final differences between antibodies. Interestingly, this affinity maturation optimizes interaction with an additional glycan at N137 Garces et al. (2015), showing that affinity maturation against glycan species is indeed possible from a germline with the characteristics to bind a GPE.

Glycan recognition by GPE targeting antibodies can be highly specific, even within closely related antibodies. Despite originating from the same putative germline, related PGT121-123 and “10-1074-like” antibodies exhibit distinct binding sensitivity to glycan modulation. PGT121-123 antibodies were more affected after PNGase F treatment, while 10-1074-like antibodies were more affected by Endo H treatment, indicating a preference of PGT121-123 for complex glycans and 10-1074 like antibodies for high-mannose species (Mouquet et al., 2012). Additionally, PGT121-123 only bound to complex, and not high-mannose, glycans on a microarray, while 10-1074-like antibodies did not bind to any glycans without protein (Mouquet et al., 2012). This evidence, in combination with evidence that GPE-Abs undergo glycan-directed affinity maturation, suggests that GPE targeting antibodies are sensitive to the glycan species. However, evaluation of the species-specificity is challenging due to the interdependence of the paratope-peptide binding, the paratope-glycan binding, and potential stabilizing or structure altering effects of the GPE in the context of the full system.

### Topological Glycoepitopes

The third group of glycoepitopes are topological glycoepitopes, which are fully glycan-dependent without a protein component (Figures 2A-III). Instead, topological glycoepitopes exist at clusters of high-mannose glycans that are spatially distributed in a distinct topological arrangement on the viral surface. The high-mannose type and the spatial arrangement of these glycans on the viral surface present distinct topological glycoepitopes that are recognized by neutralizing antibodies. The most extensively characterized example of topological glycoepitopes is that of the anti-HIV antibody 2G12 on the surface of the gp120 protein. However, topological glycoepitopes do not appear unique to HIV, and recent evidence suggests that 2G12 recognizes topological glycoepitopes on additional viruses. At the time of this writing, 2G12 glycoepitopes have been identified on Influenza H3N2 Lee et al. (2021) and SARS-CoV-2 (Acharya et al., 2020; Mannar et al., 2021).

The recognition of a topological glycoepitope which comprised exclusively of spatially distributed glycans was first reported upon discovery of the 2G12 antibody in 1996 (Trkola et al., 1996). 2G12 is a domain-exchanged (X-shaped) mAb whose format presents three glycan binding surfaces (VH-VL’, VH’-VL, and VH-VH’) (Figure 2, Right). While glycan-Ab binding events tend to be relatively low affinity, the three binding surfaces uniquely allow 2G12 to engage clusters of high-mannose N-glycans in a multivalent and high-affinity interaction (Scanlan et al., 2002; Calarese et al., 2003). The 2G12 binding interface has been investigated intensively, with multiple structures for 2G12 bound to individual glycans Sanders et al. (2013), lipooligosaccharides Stanfield et al. (2015), and also to recombinant HIV Env trimer BG505 SOSIP.664 (Seabright et al., 2020). These studies, via binding competition experiments, indicate a binding preference for terminal α1-2 linked-mannose on the Man9 d1 arm, but demonstrate binding albeit with reduced affinity to d2 and d3 arms, as well as to terminal mannose on Man1-Man8 (Sanders et al., 2002; Calarese et al., 2005; Dunlop et al., 2010; Seabright et al., 2020). These data indicate that 2G12’s glycan binding surfaces are highly accommodative of variable mannose content, which is likely a key feature of topological mAbs, given that mannose content may vary across a given topological glycoepitope.

Topological mAbs exhibit flexible valency within topological glycoepitopes. The two most recent and highest quality structural investigation of the 2G12-gp120 interaction indicate that 2G12 binds high-mannose glycans at sites N295, N332, N339, and N392 (Murin et al., 2014; Seabright et al., 2020). The investigation by Seabright *et al*. included a site-specific glycan knockout experiment for glycans within and adjacent to the 2G12 glycoepitope, finding a high degree of 2G12 binding flexibility. Knockout of any one of the four binding glycans only reduced 2G12 binding between two- and five-fold, with the greatest reduction still maintaining nanomolar binding affinity (17 nM; Seabright et al., 2020). Meanwhile, the Murin study conducted a similar glycan knockout experiment, using pseudovirus neutralization rather than binding as the readout (Murin et al., 2014). Their results indicate that glycans at sites 295, 332, and 392 are critical for pseudoviral neutralization. As is being increasingly appreciated for mAbs targeting protein epitopes, subtle epitope changes can drive critical variations in mAb function despite minor changes in mAb binding strength. This pair of binding and neutralization knock-out experiments from Seabright et al. and Murin et al. indicate that the same may be true for topological mAbs and their glycoepitopes.

The glycans within topological glycoepitopes display significant higher-order network effects. The knock-out studies performed by Seabright et al. demonstrate that N-glycans residing outside of the 2G12 binding residues affect 2G12 binding (Seabright et al., 2020). This effect is realized primarily through two mechanisms: 1) adjacent glycans contribute to the degree of N-glycan clustering and thus influence the degree of oligomannosylation at the binding glycans, and 2) adjacent glycans interact with and stabilize or destabilize the binding glycans. These properties of glycan clusters emphasize why topological glycoepitopes must be defined in the context of their glycan network, and certainly beg the question “*where do topological glycoepitopes begin and end?.*”

Topological glycoepitopes can form on quaternary structures, and this quality may contribute to the neutralizing ability of antibodies targeting topological glycoepitopes. Glycan clustering is amplified at the quaternary junctions formed by protein oligomers, such as trimeric viral proteins like SARS-CoV-2 spike protein and Influenza HA. Indeed, Lee *et al*. provide evidence that the 2G12 glycoepitope on Influenza H3N2 is quaternary and neutralizing, though the neutralizing effect may also be driven by the glycoepitope’s location proximal to the functionally-important receptor binding site (Lee et al., 2021). The 2G12 topological glycoepitope on SARS-CoV-2 is also quaternary (Acharya et al., 2020). Targeting quaternary epitopes has become increasingly appreciated as an effective neutralization strategy. Quaternary protein epitopes have been characterized for a number of viruses including HIV Robinson et al. (2010) and Zika Tharakaraman et al. (2018); Long et al. (2019), and shown to contribute to high affinity binding and potent neutralization due to inter-chain locking. Quaternary epitopes have also been shown to mediate differential and beneficial effects for mAbs targeting CD20, in which binding an epitope spanning a single tetramer vs. an epitope cross-linking adjacent tetramers results in a differential mechanism of cell killing (Marshall et al., 2017; Meyer et al., 2018). Recently, a linked biparatopic nanobody-pair that crosslinks adjacent SARS-CoV-2 Spike protein monomers was shown to have a synergistic neutralization effect as compared to the two nanobodies in combination but not linked (Koenig et al., 2021). Thus, targeting quaternary epitopes is of particular interest for imparting enhanced functionality during antibody design, and topological mAbs may be uniquely suited to target quaternary epitopes because they have no direct dependency on the protein surface at these junctions.

## Structural Characterization and Modeling of Topological Glycoepitopes

Topological glycoepitopes present unique challenges for targeting and antibody engineering. Topological glycoepitopes exist on the surfaces of a network of mobile high-mannose glycans, and so projection angles, heterogeneity, and flexibility complicate rigid structural definition that traditionally serves as the basis for most epitope-paratope complexes. Despite a previous statistical analysis of glycoproteins in the PDB providing evidence that glycan structures on homologous proteins are homogenous, the same study found that glycan orientation with respect to the protein surface was highly variable (Jo et al., 2013). Alternatively, more recent studies of the conformational heterogeneity of smaller carbohydrates (trisaccharides) demonstrate that computing the full range of conformations is required to reconcile predictions with experimental results at the atomic level (Yang et al., 2016). Dr. Robert Woods famously advised that the question “*What is the shape of my glycan*?” be rephrased to “*What shapes can the glycan adopt*?” (Woods and Tessier, 2010). For topological glycoepitopes, this line of questioning must be expanded to include additional parameters that reflect orientation and range of motion relative to the protein surface as well as interactions between glycans. Topological glycoepitope prediction, therefore, should consider the range of sampled conformations rather than a rigid snapshot of a topological mAb-topological glycoepitope complex.

This problem is most relevant for applying computationally-based rational antibody engineering to antibodies targeting topological glycoepitopes, thus requiring an *in-silico* characterization that models complex glycan-protein systems. Several webservers and computational packages exist specifically for this purpose, including GlyCAM-Web Kirschner et al. (2008), CHARMM-GUI Park et al. (2019), and Glycosylator Lemmin and Soto, (2019). In addition, several complete computational modeling programs such as Rosetta Das and Baker, (2008) include functionality for building and manipulating glycans. Each of these methods relies on a force field approach to predict the optimal (energy minimized) conformation of the system. Although different force fields contain a largely similar set of component energy terms, contributions from various factors (e.g., electrostatic vs van der Waals) are differentially weighted between models, often with the option of user-defined re-weighting which requires significant knowledge of the force field in question (Shirts et al., 2017). Therefore, when run on complex systems that include multiple interacting glycans on a protein surface, algorithms such as GLYCAM-Web and CHARMM-GUI, which rely on the GLYCAM Kirschner et al. (2008) and CHARMM Guvench et al. (2011) force fields respectively, can produce divergent results. In conclusion, unlike in other protein-protein interaction problems where static representations of protein surfaces lead to a significant number of promising docking algorithms Pagadala et al. (2017), the variability of glycoprotein structure predictions makes a single predicted set of atomic coordinates an unsuitable starting point for describing topological glycoepitopes.

Molecular Dynamics (MD) simulations, also based on force fields including those above, have been used to simulate the possible conformations of glycoproteins Lee et al. (2015), including the dynamics of the glycoepitope-containing HIV Env protein (Lemmin et al., 2017; Yang et al., 2017; Ferreira et al., 2018; Berndsen et al., 2020). These studies have elucidated insights regarding the full conformational space of Env glycans, with sampling volumes up to 50000 A^3^ for Man9 glycans Yang et al. (2017), and are able to reproduce 3D variance observed cryo-EM maps (Berndsen et al., 2020). Despite these promising results, MD simulations are computationally expensive and thus characterizing glycoepitopes across PDB using these methods remains computationally elusive, albeit highly desirable. Shortcuts to accomplish this screening task may be sufficient, such as employing glycosylation site topology as a proxy for glycan topology. Still, more advanced models may be required if rigorous relationships between antibody affinity and glycoepitope properties are to be established, especially toward the goal of rational topological antibody engineering.

## Glycoepitopes as Novel Antiviral Targets: A Perspective

Topological mAbs targeting high-mannose topological glycoepitopes have seen little development, yet offer promise as therapeutics, diagnostics, and analytical tools. Two phase one trials provide evidence that 2G12 is a clinically developable template. In the first trial, 2G12 was successfully produced in CHO cells and proven safe when co-administered in combination with another mAb (2F5) to HIV patients (Armbruster et al., 2002). In the second, 2G12 was produced in tobacco plants and shown to be safe during topical administration as a prophylactic for healthy women (Ma et al., 2015). These encouraging early data indicate that application of 2G12 to other viruses, as well as engineering efforts using 2G12 as a template, may prove fruitful.

Rational engineering efforts to expand the scope of topological mAbs may take a variety of forms. Most simply, 2G12 may be modifiable to adjust its topological footprint or glycan binding affinity and specificity, for example through hinge length alterations or paratope engineering. Second, other glycan-binding mAbs may be convertible to domain-exchanged format, which may or may not be sufficient to impart topological qualities. Investigations into the structural mechanism of 2G12 domain exchange identified that just 5-7 substitutions are required to mediate domain exchange Huber et al. (2010), though additional engineering efforts may be required to optimize placement of glycan-binding domains relative to the three topological binding surfaces. We highlight the potential for a screen searching for novel chimeric topological mAbs, *via* grafting of known glycan-binding CDRs onto the 2G12 scaffold or by induction of domain exchange *via* mutation of the cross-over residues. In particular, anti-Bacterial glycan-targeting mAbs which tend to have desirable qualities—flexible recognition of multiple species due to targeting of “minimal” glycoepitopes Rollenske et al. (2018)—as well as diagnostic microarrays, which have already characterized sets of mAbs and their glycan specificities Campanero-Rhodes et al. (2020), may serve as logical starting points. As described earlier, computational methods to predict glycan cluster topology and variability would greatly enhance these efforts. Third, such optimizations may also investigate the dimeric form of 2G12, which was shown to increase neutralization potency against HIV by 50-80-fold (West et al., 2009). Subsequent structural investigations suggest that enhanced potency is driven by increased flexibility Wu et al. (2013), and further functional investigation found that the dimeric 2G12 format also enhances ADCC (Klein et al., 2010).

Pursuing topological glycoepitope and topological mAb research efforts could prove particularly useful for eventual rapid response deployment. In one hypothetical scenario, with precedent according to the previously described Flu “glycan clock,” the emergence of an Influenza H1N1 or SARS-CoV-2 variant with a novel N-glycan site obscuring an immunodominant epitope adjacent to the H1N1 RBS or SARS-CoV-2 RBD might result in a sudden increase in virulence in vulnerable populations. In such a scenario, pre-existing development work on 2G12 or other topological mAb rational engineering might prove highly beneficial for rapid response, perhaps *via* prophylactic administration to vulnerable populations. The need for template engineering, rather than use of WT 2G12, is highlighted by the early data presented earlier Acharya et al. (2020) demonstrating that WT 2G12 glycoepitopes exist on SARS-CoV-2 but do not facilitate neutralization, likely due to their location distal from the spike protein receptor binding domain (RBD). If additional N-glycan sites were to be evolved within the SARS-CoV-2 RBD, a topological mAb might need to be engineered to display a preference for the RBD glycan topology rather than the RBD-distal topological glycoepitope.

In addition to their therapeutic value, topological mAbs could be employed as diagnostic and analytical tools. Due to the difficulty of characterizing glycans and glycoproteins, glycan arrays have historically been deployed to assay samples using sets of glycan binding agents with well-characterized targets. In example, a straightforward application of 2G12 toward this purpose is its use to characterize gp120 or HIV immunogens for glycan presentation (Doores et al., 2013). This has been a mainstay approach to HIV vaccine development, though glycan mimicry alone has thus far fallen short of generating neutralizing responses (Seabright et al., 2019). A similar approach leveraging topological binding to quaternary epitopes could be taken to assay for proper oligomerization of glycoproteins that only display functional topological epitopes in their oligomerized state, for example on Influenza H3N2. Such an analytical tool could prove useful for validating seasonally-adjusted influenza vaccines, integrating well with existing mass spectrometry-based approaches. In conclusion, we believe that topological glycoepitopes offer fruitful therapeutic, diagnostic, and analytical opportunities, and hope that this perspective motivates further study of this interesting epitope-paratope interaction.
